# Community-Engaged Research of Older Pacific Islander Adults: Results From the PIHS and the NHPI NHIS

**DOI:** 10.1093/geroni/igaa057.3098

**Published:** 2020-12-16

**Authors:** Sela Panapasa

**Affiliations:** University of Michigan, University of Michigan, Michigan, United States

## Abstract

Despite the well-established need to measure and address the growing issue of disparities among older minority populations, little is known about the prevalence and correlates of disability, morbidity, and mortality among older US Pacific Islander adults. This paper discusses culturally appropriate approaches for conducting evidence-based research on a representative probability sample of older Native Hawaiian and Pacific Islander adults interviewed as part of the Pacific Islander Health Study and Native Hawaiian and Pacific Islander National Health Interview Survey. Important strategies that increase response rates and respondent participation when engaging this multi-ethnic and culturally diverse special population in research are highlighted. The model describes successful methodologies that combine CBPR approaches of community engagement with more traditional survey design methods. The findings from this work emphasizes the importance of representative data on hard-to-survey populations to illustrate granular differences in health outcomes within underrepresented populations that are not reflected in national health surveys.

